# Efficacy and Safety of Different Intravenous Glucocorticoid Regimens in the Treatment of Graves' Ophthalmopathy: A Meta-Analysis

**DOI:** 10.1155/2021/9799274

**Published:** 2021-07-12

**Authors:** Jing Chen, Nuo Xu, Huilan Sun, Gang Chen

**Affiliations:** ^1^Department of Ophthalmology, Fujian Provincial Hospital, Fuzhou, Fujian, China; ^2^The Shengli Clinical Medical College, Fujian Medical University, Fuzhou, Fujian, China; ^3^Department of Endocrinology, Fujian Provincial Hospital, Fuzhou, Fujian, China; ^4^Fujian Provincial Key Laboratory of Medical Analysis, Fujian Academy of Medical Sciences, Fuzhou, Fujian, China

## Abstract

**Purpose:**

The intravenous glucocorticoid (iv GC) represents the mainstay of therapy for Graves' ophthalmopathy (GO), but uncertainty remains concerning the optimal regimen. Although the European Group on Graves' Orbitopathy (EUGOGO) regimen has been commonly employed, evidence for its superiority to other regimens is still lacking. The aim of this meta-analysis was to compare the efficacy and safety of the EUGOGO regimen with higher-dose regimens in the management of GO.

**Methods:**

A systematic review and meta-analysis of randomized controlled trials (RCTs) and cohort studies comparing the EUGOGO regimen with higher-dose regimens was conducted. PubMed, Embase, and Web of Science databases were searched for relevant studies. The efficacy outcomes were response rate, change in clinical activity score (CAS), rate of proptosis improvement, and retreatment rate. The safety outcome was the incidence of adverse events.

**Results:**

In the five included eligible trials, 136 participants in the EUGOGO regimen and 177 participants in higher-dose regimens were evaluated. Compared with the EUGOGO regimen, higher-dose regimens had no beneficial effect on the response rate, change of CAS, rate of proptosis improvement, and retreatment rate (OR: 1.3; 95% CI: 0.36–4.65; SMD: –0.04; 95% CI: –0.54, 0.45; OR: 0.79; 95% CI: 0.44–1.44; OR: 0.87; 95% CI: 0.27–2.77). For the incidence of adverse events, the results also showed no significant difference between the 2 groups (OR: 1.14; 95% CI: 0.62–2.09).

**Conclusion:**

The current evidence showed that the efficacy of the EUGOGO regimen was comparable with higher-dose regimens. Since there was no significant difference in the incidence of adverse events between the two regimens, appropriate selection of patients and careful monitoring were required in both regimens. More well-designed, large-scale, and longer follow-up period studies were needed to further verify the finding of this analysis.

## 1. Introduction

Graves' ophthalmopathy (GO) is an orbital autoimmune disorder [1], which is characterized by periorbital edema, restrictive strabismus, and proptosis [2]. This disease has a relatively high incidence and remains one of the most challenging diseases to manage owing to its complex and poorly understood pathogenesis [3, 4]. Although physicians and scientists continue their efforts to optimize treatment recommendations [5], most treatments for active, moderate-to-severe GO remain suboptimal [6].

Glucocorticoids have been widely used to modulate the immune system and reduce inflammation in a myriad of medical conditions. Several studies have demonstrated the effectiveness of glucocorticoids in GO treatment [7, 8], but the regimen of glucocorticoid, ranging from the drug dosage and the administration route to the course of treatment, varied among different studies. Among them, the commonest and recommended regimen is a 4.5 g cumulative dose subdivided into 12 weekly infusions (EUGOGO regimen) [9]. In terms of its efficacy, the results showed that about 20–30% of patients were unresponsive or poorly responsive and up to 20% suffered reactivation after completing the treatment [10, 11]. Therefore, some researchers suggested that higher doses might be able to achieve better results. Recently, various studies have compared the EUGOGO regimen with higher-dose regimens on the efficacy and safety of GO treatment, but the results remain controversial [12]. Since the evidence-based demonstration of the optimal therapeutic regimen is still lacking, we conducted a meta-analysis to compare the benefits and harms of the EUGOGO regimen with higher-dose regimens for the treatment of GO.

## 2. Materials and Methods

### 2.1. Search Strategy

This meta-analysis was conducted according to the Preferred Reporting Items for Systematic Reviews and Meta-Analysis (PRISMA) guidelines [13]. Two reviewers (Jing Chen and Huilan Sun) performed a systematic search in the databases PubMed, Embase, and Web of Science (up to September 2020) to identify studies that compared the EUGOGO regimen with higher-dose regimens. We used the following search terms: “glucocorticoid” or “methylprednisolone” or “corticosteroid” or “steroid” or “prednisone” and “Graves ophthalmopathy” or “endocrine ophthalmopathy” or “dysthyroid ophthalmopathy” or “thyroid ophthalmopathy” or “thyroid associated ophthalmopathy” or “Graves orbitopathy” or “endocrine orbitopathy” or “thyroid orbitopathy” or “thyroid associated orbitopathy” or “Graves eye disease” or “thyroid eye disease.” In addition, the reference lists of relevant reviews and eligible studies were also searched for additional studies. There was no language restriction.

### 2.2. Study Selection

Studies fulfilling the inclusion criteria were included: (1) patients with moderate-to-severe GO; (2) trials comparing the EUGOGO protocol with higher-dose regimens in GO treatment; (3) one or more of the outcome variables be reported, including response rate, change of CAS, rate of proptosis improvement, retreatment rate, and incidence of adverse events. Studies were excluded as follows: (1) abstracts, case reports, letters, reviews, or nonclinical studies; (2) studies with insufficient data for calculating the results; (3) duplicated publications.

### 2.3. Data Extraction and Quality Assessment

Two reviewers (Jing Chen and Huilan Sun) independently evaluated and extracted the eligible studies. Any discrepancies were resolved by consulting with a third reviewer (Gang Chen). The efficacy outcomes were the response rate, change of CAS, rate of proptosis improvement, and retreatment rate. The safety outcome was the incidence of adverse events. For each included study, the following data were extracted: response rate, change of CAS, rate of proptosis improvement, retreatment rate, and incidence of adverse events. In addition, we also recorded authors, published year, country, study design, sample size, age, gender, disease activity and severity, treatment regimen, length of follow-up, and quality of each trial. The quality of included randomized controlled trials was assessed by the Jadad scale, and cohort studies were assessed by the Newcastle-Ottawa scale (NOS). Studies with Jadad score greater than 3 or NOS score greater than 6 were defined as high-quality studies [14].

### 2.4. Statistical Analysis

The meta-analysis was performed using Stata 12.0 software. We calculated standard mean difference (SMD) with 95% confidence intervals (CIs) for continuous variables and odds ratios (ORs) with 95% CIs for dichotomous variables. Two-sided *P* < 0.05 was considered to be statistically significant. Heterogeneity was estimated using the *χ*^2^-based *Q* statistic and *I*-squared (*I*^2^) test. It was considered that studies lacked the significant heterogeneity when *P* value > 0.10 and *I*^2^ < 50%; then, the fixed-effect model was applied. Otherwise, the random-effect model was used.

## 3. Results

### 3.1. Study Characteristics

**Figure 1:**
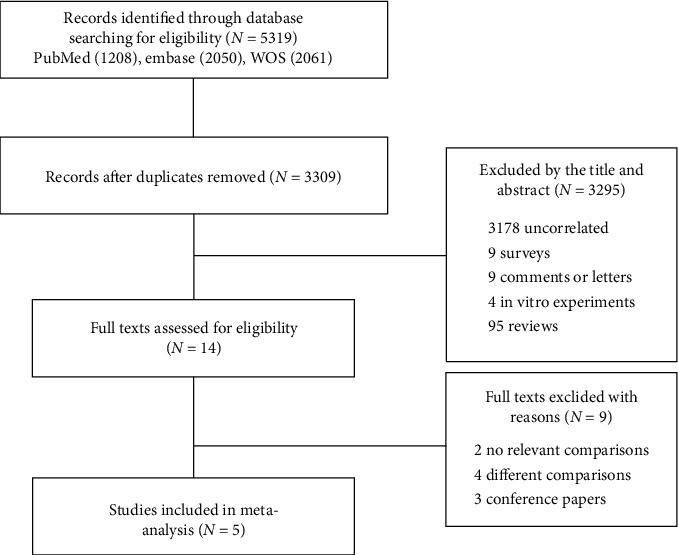
Flow diagram of the study identification and selection process.

The screening and selection processes are shown in Figure 1. A total of 5319 articles were identified after initial search. After duplicated studies were removed, 3309 articles were left for title and abstract screening; then, 3295 articles were excluded because they were not meeting the inclusion criteria. Subsequently, the remaining 14 articles were identified for full-text review. Finally, 5 eligible articles were available for inclusion. Four trials [15–18] compared two different iv GC regimens. One trial [10] compared three different regimens (low dose vs. middle dose vs. high dose), and data from both middle- and high-dose groups were included in this study. The doses of the control group were all greater than or equal to 4.5 g, so they were classified as the higher-dose regimen. The characteristics of selected studies are summarized in Table 1. Overall, 136 participants in the EUGOGO regimen and 177 participants in the high-dose regimen were evaluated. All participants were rated with the severity moderate to severe; the mean age was from 41.8 to 46.8 years. The single doses were among 0.25 g to 1 g, cumulative doses ranged from 4.5 g to 18 g, dosing interval ranged from 1 week to 4 weeks, and treatment course ranged from 4 weeks to 24 weeks. The mean follow-up ranged from 11 weeks to 24 weeks.

### 3.2. Efficacy Outcomes

**Figure 2:**
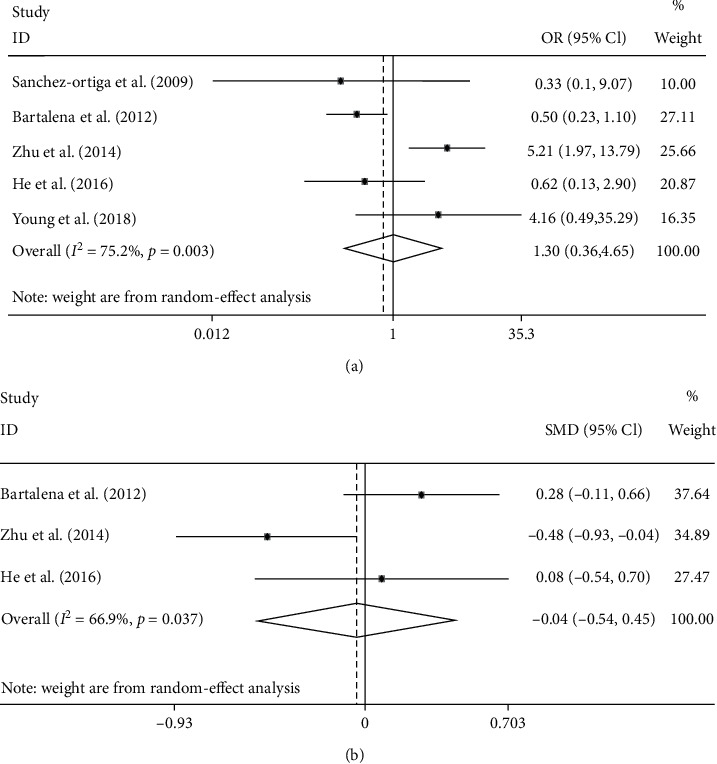
Forest plot of the response rate (a) and change of CAS (b), EUGOGO regimen vs. higher-dose regimen.

**Figure 3:**
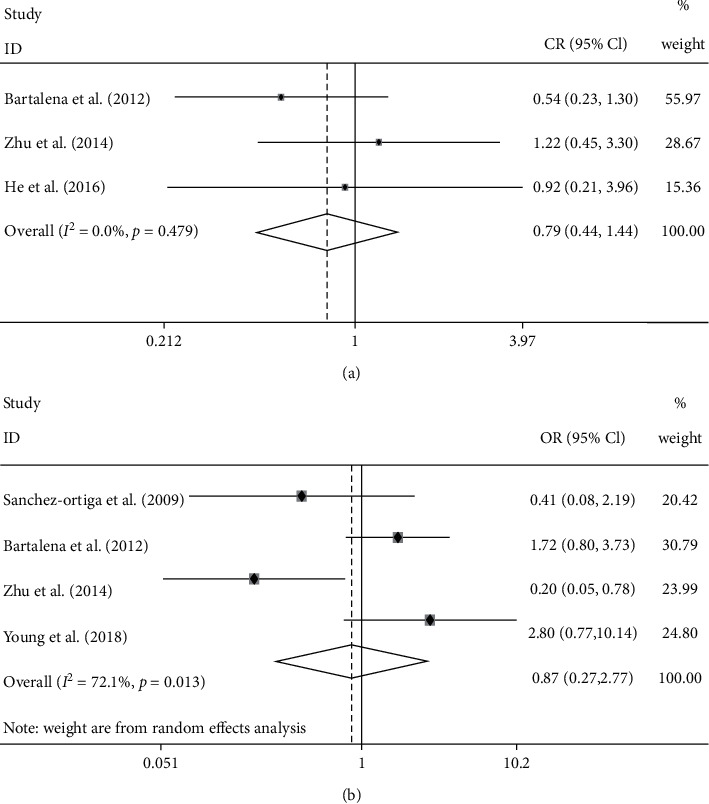
Forest plot of the rate of proptosis improvement (a) and retreatment rate (b), EUGOGO regimen vs. higher-dose regimen.

Response rate was evaluated in all 5 studies; the result showed no significant difference between the 2 groups (Figure 2(a)) (OR: 1.3; 95% CI: 0.36–4.65; random model; *I*^2^: 75.2%). Three trials provided data on change of CAS, and there was no significant difference existing between the 2 groups (Figure 2(b)) (SMD: −0.04; 95% CI: −0.54, 0.45; random model; *I*^2^: 69.6%). Three studies measured the rate of proptosis improvement, and the pooled result showed no significant difference between the 2 groups (Figure 3(a)) (OR: 0.79; 95% CI: 0.44–1.44; fixed model; *I*^2^: 0.0%). In view of the retreatment rate, the pooled result of 4 trials showed no significant difference between the 2 groups (Figure 3(b)) (OR: 0.87; 95% CI: 0.27–2.77; random model; *I*^2^: 72.1%).

### 3.3. Safety Outcome

**Figure 4:**
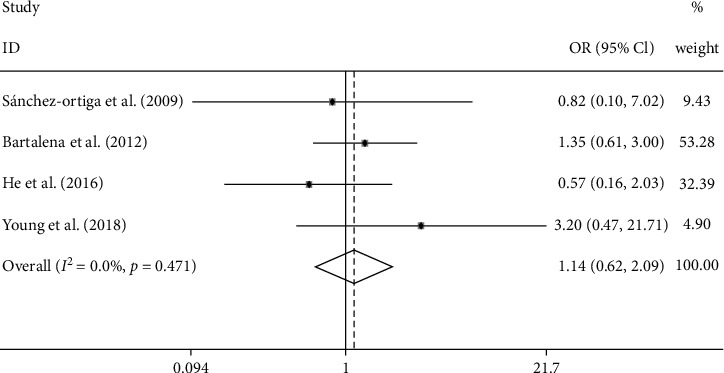
Forest plot of the incidence of adverse events, EUGOGO regimen vs. higher-dose regimen.

Among the 5 included studies, 4 studies reported the incidence of adverse events, and there was no significant difference between the 2 groups (Figure 4) (OR: 1.14; 95% CI: 0.62–2.09; fixed model; *I*^2^: 0.0%).

## 4. Discussion

The optimal iv GC regimen for GO treatment has been the topic of more recent investigations [19], but the results are inconsistent and inconclusive. Since the evidence-based demonstration of the optimal therapeutic regimen is still lacking [12], we reviewed the published studies and conducted a meta-analysis to derive a more precise estimation of the ideal treatment regimen. Our findings suggested that the efficacy of the EUGOGO regimen was comparable with higher-dose regimens.

The purposes of medical treatment for moderate-to-severe GO are to reduce disease activity, improve muscle involvement, decrease optic nerve compression if present, and, ultimately, decrease the subsequent need of rehabilitative surgery [12]. The rationale of GC treatment stems from its anti-inflammatory and immunosuppressive effects. Their effects are exerted through genomic and nongenomic actions [11]. Genomic actions ultimately cause increased synthesis of anti-inflammatory proteins and decreased synthesis of proinflammatory proteins [20, 21]. The nongenomic actions related to physicochemical changes of the cellular membrane or interaction with membrane-bound receptors, thereby leading to cellular membrane stabilization [22]. The beneficial effect of iv GC treatment on soft tissue swelling, visual acuity, and ocular motility has been verified. However, the impact of administered dose and therapy schedule have not been fully assessed yet. Although the responsive rate of the currently recommended EUGOGO regimen is assumed approximately 80% [10, 11, 16], some researchers argued that the protocol was advocated to reduce significant life-threatening complications. While this was safer, its efficacy however was much limited [11]. Also, several studies suggested that dose defines the strength of beneficial effects, and the higher-dose regimen might achieve a more rapid and effective immune suppression, eventually increasing the response rate [9, 23]. However, more trials suggested otherwise. He et al. found no significant difference in the response rate between the higher-dose regimen (a total dose of 6 g over 3 months) and EUGOGO regimen [17]. A similar finding was reported by Ueda-Sakane et al., in which no significant difference in ophthalmic parameters reflecting treatment efficacy was found, even a higher-dose regimen (cumulative dose: 9–12 g) was used [24]. Our study confirmed again that the higher-dose regimen did not show extra effects on response rate when compared with the EUGOGO regimen.

Glucocorticoids are rapid, potent, and highly effective in inactivating GO. Using CAS as a tool for assessing inflammation, inactivation of GO (final CAS ≤ 2/7) has been reported in about 60% of cases in 9 randomized studies and 90% of cases in 13 nonrandomized studies [11]. In recent years, some studies suggested that higher doses of GC might be able to decrease inflammation better [17, 23], but evidence from our study showed that CAS behaved similarly in both regimens. Other studies also reported similar results. Young et al. reported no significant difference between the high-dose protocol (19 g) and the modified EUGOGO protocol (4.5 g) in both CAS and ITEDS system scores [18]. Bartalena et al. found that the difference in the rate of CAS decrease was similar between the high-dose (HD) group (7.47 g) and middle-dose (MD) group (4.98 g). However, the inactive rate (CAS ≤ 2) was higher in MD patients (65%) compared with HD patients (60%) [10].

Relapses of active GO are a rather common and tough problem after iv GC therapy, and a significant number of patients who respond to glucocorticoids initially might experience disease reactivation after therapy withdrawal [25]. Therefore, the need for retreatment is another important criterion to evaluate treatment efficacy [16], and a better understanding of potential strategies to reduce the risk of relapse is warranted [12]. Several studies suggested that the dose and treatment schedules might have an impact on the relapse rate [15–17]. Sánchez-Ortiga et al. reported that compared with the higher-dose regimen, a cumulative dose of 4.5 g (12 weeks) regimen appears to be associated with fewer relapses [15]. In another study, Zhu et al. found that, with the same administration dose, the weekly protocol had fewer retreatment events and prolonged retreatment-free survival, compared with the daily protocol [16]. He et al. reported that compared to the weekly regimen group, lower recurrence rates were found in the monthly regimen group [17]. In contrast to these studies, our result suggested that there was no significant difference between the two regimens.

What were the reasons for the comparable efficacy regarding CAS reduction, response rate, and retreatment rate between the two regimens? Firstly, it might be that the dose used in the EUGOGO regimen could achieve the same effect as the higher-dose regimen. Secondly, GO is a single flare of the autoimmune process [9], and the severity of illness might fluctuate if the interval between any two cycles is greater than 1 week [17]. Thus, the weekly protocol might achieve greater, more sustained suppression of local inflammation and prolonged retreatment-free survival [16]. In our meta-analysis, two studies (He et al. and Young et al.) in the higher-dose group had drug intervals of more than 2 weeks; even if higher doses might be able to achieve slightly better results, this part of the advantage might be offset.

In terms of proptosis, although iv GC was considered as one of the best treatment strategies in a network meta-analysis [26], its effect in proptosis improvement is marginal with the reduction of proptosis ranging from 0.6 mm to 2 mm [9, 10, 27]. Bartalena et al. came to a conclusion that, even using the highest dose, the average decrease of proptosis was less than 1 mm. Furthermore, high dose and middle dose led to almost the same result [10]. As mentioned above, our study also found no significant difference of the rate of proptosis improvement between the two regimens. The underlying mechanism is yet to be completely elucidated; it might be that the patient's orbital remodeling and eventual fibrosis occur very early in the course of the disease, so there is no hope that whatsoever medical treatment be effective if these changes have taken place [28].

Although effective, GC treatment is not devoid of adverse events [12, 29]. Their toxicity is related to preexisting disease, dose, and treatment schedule [11] and remained one of the most common causes of iatrogenic illness [30]. Numerous studies have demonstrated that adverse events seem to be dose dependent [9, 27, 31, 32]. However, in our study, no such association was noted. The possible reason is that although major adverse events were dose related, they occur more frequently with doses over 8 g [33]. On the contrary, minor side effects were more common irrespective of the GC dose [10]. In our meta-analysis, the most dosage in the higher-dose group did not exceed 8 g, with the exception of one study that used a dose of 18 g. Therefore, our study might be underpowered to detect the difference. It was worth noting that even though severe adverse events were more common using high doses, low-dose therapy was not devoid of serious risks. Thus, appropriate selection of patients and careful monitoring were required at any dose [34].

There were some limitations in this meta-analysis. The first one is the potential publication bias. It was possible that some unpublished studies were inevitably missed. The second limitation was the small number of included studies, and some studies only reported part of outcomes. The limited sample size might prevent robust conclusions. Furthermore, the follow-up time for all studies was shorter than 6 months, which might influence the evaluation of efficacy and safety outcomes.

## 5. Conclusion

Our meta-analysis showed that the current iv GC regimen was diverse and compared the efficacy and safety of different iv GC regimens in the management of GO for the first time. Our analysis of current evidence revealed that the efficacy of the EUGOGO protocol was comparable with the higher-dose regimen. It should be noted that since there was no significant difference in the incidence of adverse events between the two groups, appropriate selection of patients and careful monitoring were warranted at any dose. In addition, due to the inherent limitations of the included studies, more well-designed, large-scale, and longer follow-up duration studies were needed to further verify the finding of this analysis.

## Figures and Tables

**Table 1:** Baseline characteristics of included trials.

Study	Year	Location	Study	*N*	Age	Sex (M/F)	Stage	EUGOGO regimen				Higher-dose regimen					
								Intervention	Dose (g)	Course (wk)	*N*	Intervention	Dose	Course (wk)	*N*	Follow-up (wk)	Quality
Sánchez-Ortiga et al. [15]	2009	Spain	RDS	24	45.3	4/20	Active, moderate-severe	0.5 g/wk^*∗*^6 wk and then 0.25 g/wk^*∗*^6 wk	4.5	12	13	4 cycles of 15 mg/kg and then 4 cycles of 7.5 mg/kg	90 mg/kg	16	11	11	H
Bartalena et al. [10]	2012	Europe	RCT	159	NA	49/110	Active, moderate-severe	0.54 g/wk^*∗*^6 wk and then 0.29 g/wk^*∗*^6 wk	4.98	12	54	0.83 g/wk^*∗*^6 wk and then 0.415 g/wk^*∗*^6 wk	7.47 g	12	52	24	H
Zhu et al. [16]	2014	China	RCT	80	46.8	34/46	Active, moderate-severe	0.5 g/wk^*∗*^6 wk and then 0.25 g/wk^*∗*^6 wk	4.5	12	39	(0.5 g/daily^*∗*^3 days)^*∗*^2 wk and then (0.25 g/daily^*∗*^3 days)^*∗*^2 wk	4.5 g	4	41	12	H
He et al. [17]	2016	China	RCT	40	41.8	14/26	Moderate-severe	0.5 g/wk^*∗*^6 wk and then 0.25 g/wk^*∗*^6 wk	4.5	12	18	(0.5 g/daily^*∗*^3 days)^*∗*^3 months	6 g	12	22	13	H
Young et al. [18]	2018	Singapore	PCS	63	43.1	32/31	Active, moderate-severe	0.5 g/wk^*∗*^6 wk and then 0.25 g/wk^*∗*^6 wk	4.5	12	12	(1 g/daily^*∗*^3 days)^*∗*^6 months	18 g	24	51	24	H

RDS: retrospective descriptive study; RCT: randomized controlled trial; PCS: prospective cohort study.

## Data Availability

The datasets used and/or analyzed during the current study are available from the corresponding author upon reasonable request.
